# Case Report: A rare adult multisystem Langerhans cell histiocytosis involving the skin, thyroid, jaw-periodontal region, and bone: diagnostic challenges and complementary PET/CT–SPECT/CT findings

**DOI:** 10.3389/fonc.2026.1784088

**Published:** 2026-02-27

**Authors:** Longchuan Zhuang, Kexu Chen, Xiaokun Qi, Jie Li, Song Qin, Hua Yu, Minggang Yuan

**Affiliations:** 1 Yantai Institute of Coastal Zone Research, Chinese Academy of Sciences, Yantai, Shandong, China; 2 Department of Nuclear Medicine, Qingdao Central Hospital, University of Health and Rehabilitation Sciences, Qingdao, Shandong, China; 3 Yantai Key Laboratory of Nuclear Safety for Marine Ecological Environment, Yantai, Shandong, China; 4 Shandong Key Laboratory of Coastal Zone Environmental Processes and Ecological Security, Yantai, Shandong, China; 5 Affiliated Qingdao Third People’s Hospital, Qingdao University, Qingdao, Shandong, China

**Keywords:** ^18^F-FDG PET/CT, adult multisystem disease, bone scintigraphy with SPECT/CT, cutaneous involvement, Langerhans cell histiocytosis

## Abstract

Langerhans cell histiocytosis (LCH) is a rare clonal myeloid neoplasm with prominent inflammatory features and heterogeneous manifestations in adults, often leading to diagnostic delay. We report a 32-year-old man who presented with a two-year history of recurrent ulcerative intertriginous skin lesions refractory to empirical treatment for presumed inflammatory or infectious dermatoses. Biopsy from the ulcer edge showed atypical histiocytoid cells with characteristic nuclear grooves and numerous eosinophils; immunohistochemistry was positive for CD1a, S-100, and Langerin with a Ki-67 index of ~40%, confirming cutaneous LCH. Baseline ^18^F-FDG PET/CT (August 2022) demonstrated multisystem disease with extensive cutaneous involvement and imaging-suggestive involvement of multiple extracutaneous sites, including multifocal osseous lesions, the thyroid, gastrointestinal tract, lymph nodes, lung (cystic lesions), and an intramuscular nodule. After six cycles of cytarabine-based induction chemotherapy, follow-up PET/CT (April 2023) showed near-complete metabolic remission with only residual low-grade uptake in the perineal skin and maxillomandibular/gingival region. In contrast, ^99m^Tc-MDP whole-body bone scintigraphy with SPECT/CT performed one year later (July 2024) revealed persistent multifocal tracer uptake, most prominent in the craniofacial skeleton and long bones, with corresponding CT changes suggestive of ongoing remodeling. This case highlights the importance of early biopsy of persistent unexplained ulcerative intertriginous lesions in adults to expedite systemic staging, and demonstrates the complementary roles of serial ^18^F-FDG PET/CT and bone scintigraphy with SPECT/CT in longitudinal assessment of osseous disease in adult multisystem LCH.

## 1 Introduction

**Figure 1:**
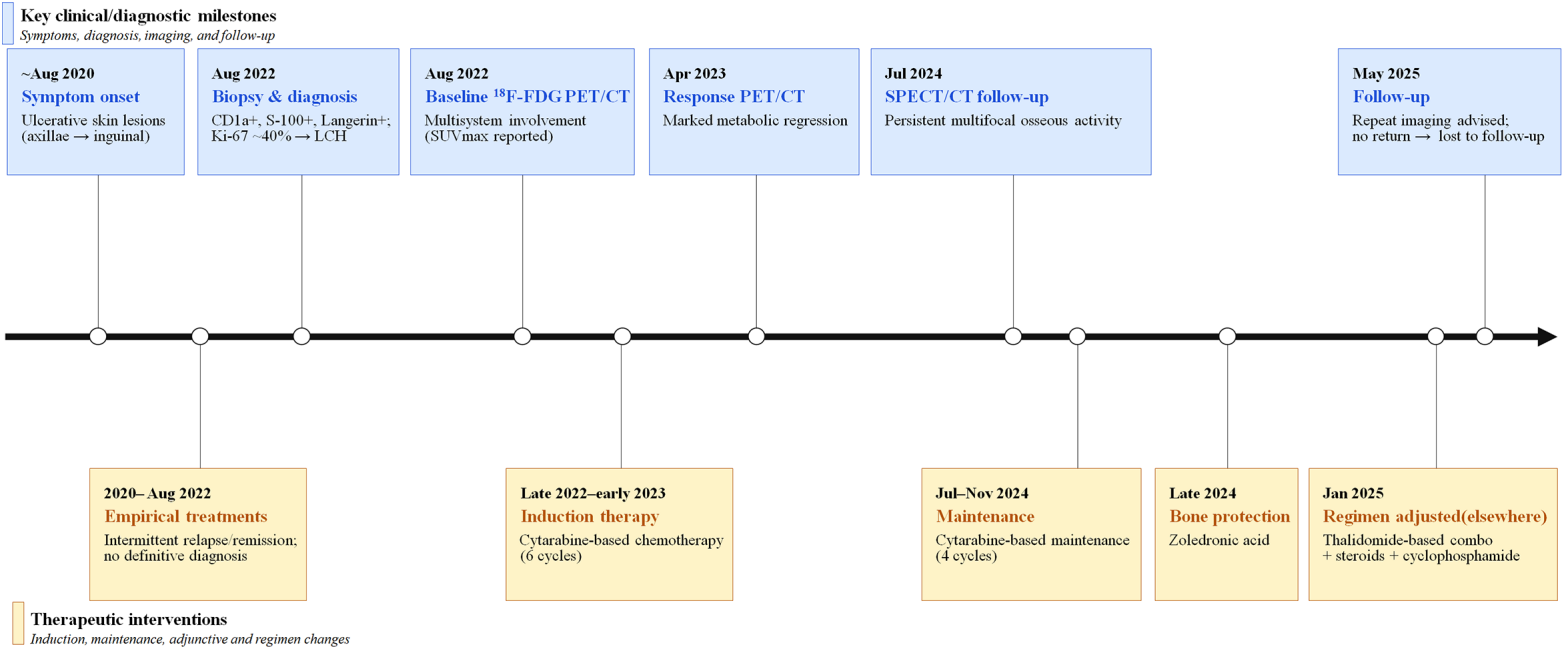
Timeline of the patient’s clinical course, including diagnostic confirmation, serial imaging assessments, and treatment milestones.

Langerhans cell histiocytosis (LCH) is now classified as a rare clonal myeloid neoplasm with prominent inflammatory features, commonly driven by MAPK pathway activation (1, 2). The incidence is approximately 5 cases per million children and ~1 case per million adults (3). To date, there is no universally accepted, evidence-based first-line standard of care for adult LCH, and current treatment recommendations are largely derived from retrospective series and expert consensus rather than randomized trials (4). This therapeutic uncertainty is particularly relevant in adults with multisystem LCH, which frequently manifests with nonspecific symptoms, resulting in prolonged diagnostic delays (5). Within this already rare entity, pulmonary and pituitary involvement are relatively well characterized (6, 7), whereas extensive and atypical skeletal involvement—particularly affecting craniofacial and other unusual flat bones—has been reported only sporadically and remains insufficiently documented. Here, we report a rare case of adult multisystem Langerhans cell histiocytosis (LCH) in a 32-year-old man who experienced a two-year diagnostic delay due to recurrent ulcerative intertriginous lesions despite multiple empirical treatments, with the diagnosis ultimately established by ulcer-edge biopsy and immunophenotyping. Subsequent serial imaging demonstrated extensive and atypical skeletal and oral involvement, with complementary longitudinal findings between ^18^F-fluorodeoxyglucose positron emission tomography/computed tomography (^18^F-FDG PET/CT) and whole-body bone scintigraphy with single photon emission computed tomography/computed tomography (SPECT/CT), underscoring the value of multimodality imaging in assessing adult multisystem LCH (**Figure 1**).

## 2 Case presentation

A 32-year-old male presented to our institution with a two-year history of recurrent ulcerative skin lesions initially involving the bilateral axillae and later the inguinal region, with intermittent partial remission and relapse. The onset of symptoms occurred during a period of significant occupational stress. Before referral to our institution, the patient had received multiple empirical treatments for presumed inflammatory or infectious skin conditions, without a definitive diagnosis. During the disease course, he remained afebrile, with preserved appetite and sleep, normal bowel and bladder habits, and no significant weight loss.

The patient had been previously healthy, with no history of diabetes mellitus, hypertension, coronary artery disease, chronic infections (including tuberculosis or viral hepatitis), major trauma, surgery, blood transfusion, or known immunodeficiency. He denied any drug or food allergies, and routine vaccinations were up to date. He reported no history of long-term residence in endemic areas, occupational exposure to dust, toxic chemicals, or ionizing radiation, and denied consumption of raw fish or meat. He was a lifelong non-smoker and denied alcohol use. There was no history of significant psychological trauma, high-risk sexual behavior, or substance abuse. He was married with one healthy child, and there was no family history of hereditary disorders, autoimmune diseases, or malignancies.

Initial laboratory testing showed mild inflammatory activity (C-reactive protein 7.22 mg/L). A borderline anti-Sm reactivity was noted on an ANA/ENA panel; in the absence of clinical features suggestive of systemic autoimmune disease, this isolated finding was interpreted cautiously. Additional tests showed nonspecific hematologic and biochemical abnormalities, including mild anemia and a cholestatic pattern of liver biochemistry.

**Figure 2:**
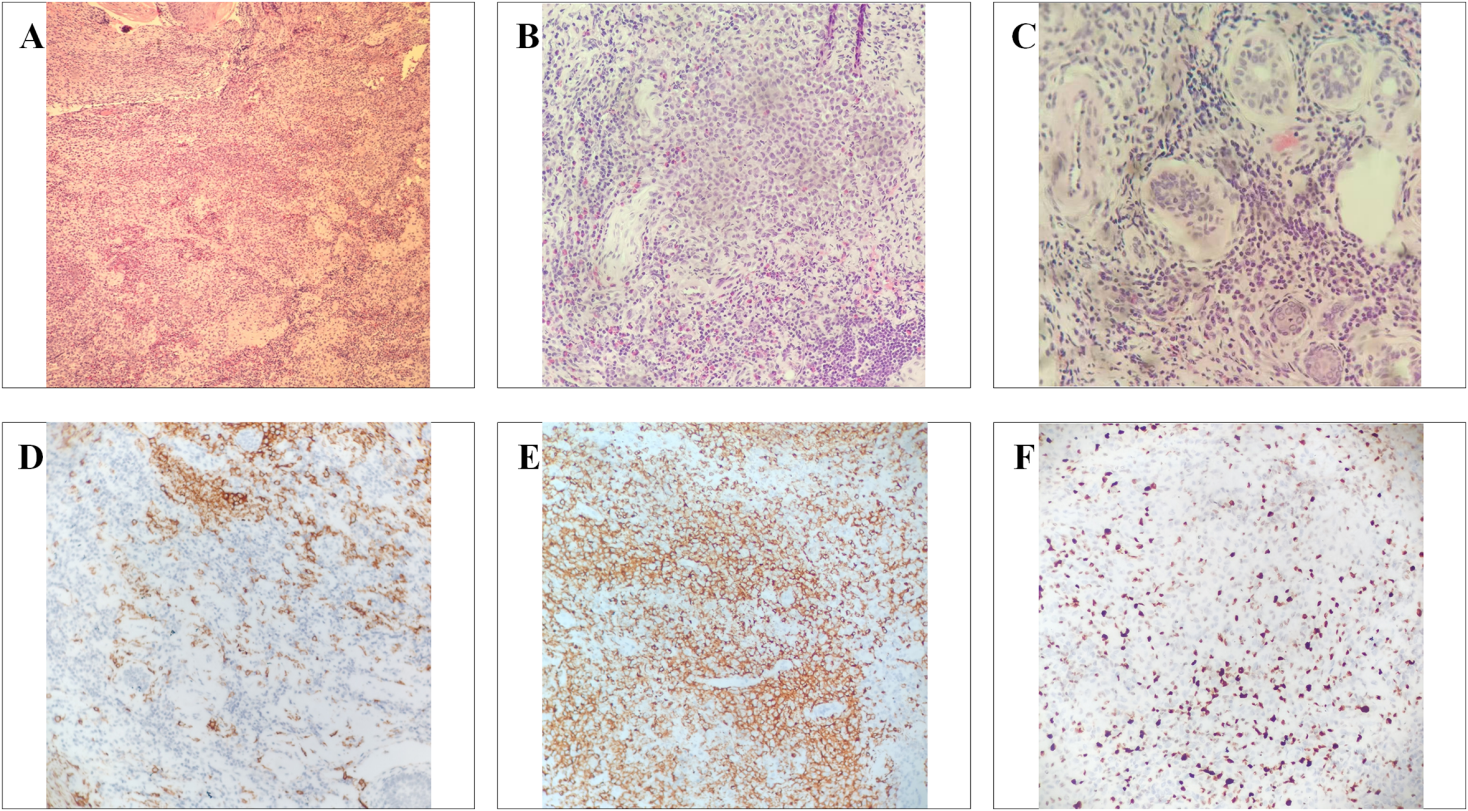
Histopathology and immunophenotype confirming Langerhans cell histiocytosis. **(A–C)** Hematoxylin and eosin (H&E) staining at low (×100), intermediate (×200), and high magnification (×400) demonstrates a dermal infiltrate of atypical histiocytoid cells with pale cytoplasm and characteristic nuclear grooves, accompanied by numerous eosinophils and mixed inflammatory cells. **(D)** Immunohistochemistry shows diffuse membranous positivity for CD1a in lesional cells. **(E)** Lesional cells are positive for Langerin. **(F)** Ki-67 labeling index is approximately 40%, indicating an increased proliferative activity.

Persistent ulceration in the inguinal region prompted a comprehensive diagnostic evaluation at our institution, including histopathological examination. Hematoxylin–eosin sections from the ulcer edge demonstrated an infiltrative dermal proliferation of atypical cells with abundant pale cytoplasm and irregular grooved nuclei, arranged in clusters and nodules, accompanied by numerous eosinophils and mixed inflammatory cells. Subsequent immunohistochemistry demonstrated positivity for CD1a, S-100, and Langerin, with a Ki-67 proliferation index of approximately 40%, establishing the diagnosis of Langerhans cell histiocytosis (LCH) (**Figure 2**).

As part of systemic staging at baseline, bone marrow examination showed hypocellular marrow (~30%) with preserved myeloid–erythroid maturation and rare megakaryocytes; reticulin fibrosis was MF-0. Conventional cytogenetics revealed a normal male karyotype [46, XY (20)]. Testing for BRAF p.V600E was negative.

**Figure 3:**
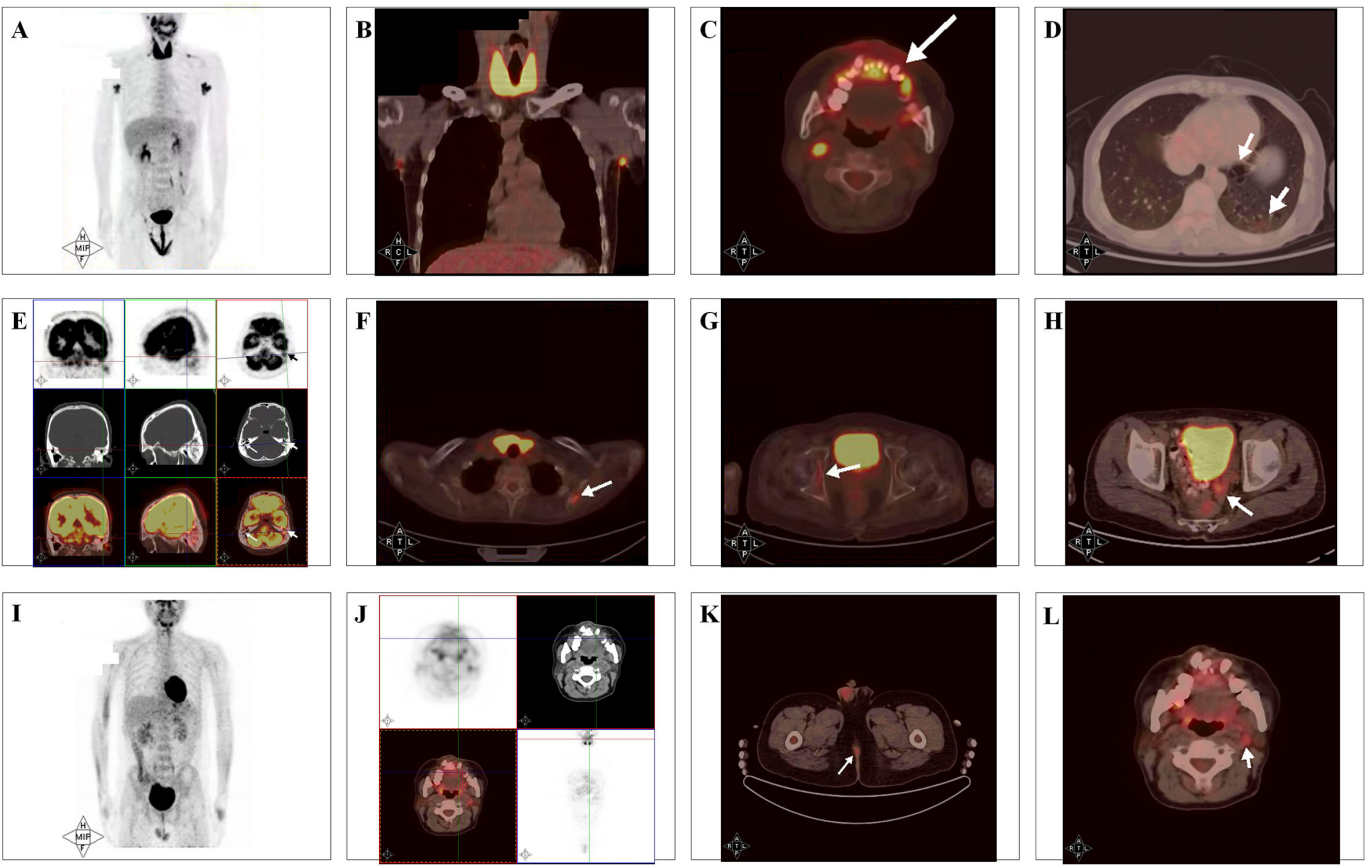
Baseline and post-induction ^18^F-FDG PET/CT in adult multisystem Langerhans cell histiocytosis. **(A–H)** Baseline ^18^F-FDG PET/CT (August 2022): whole-body MIP **(A)** and representative FDG uptake in the thyroid and axillary skin **(B)**, maxillomandibular/gingival region **(C)**, pulmonary lesions (cystic lesion with mild uptake and a non–FDG-avid solid nodule) **(D)**, temporal/mastoid region **(E)**, scapula (lytic FDG-avid lesion) **(F)**, acetabulum **(G)**, and sigmoid colon **(H)**. **(I–L)** Post-induction ^18^F-FDG PET/CT (April 2023): whole-body MIP showing markedly decreased disease burden **(I)**, with residual low-grade uptake in the maxillomandibular/gingival region (SUV max 5.3; baseline 6.1) **(J)** and perineal cutaneous lesions (SUV max 3.7; baseline 10.4) **(K)**; mild cervical nodal uptake was favored to be reactive **(L)**. Arrows indicate representative findings.

Initial ^18^F-FDG PET/CT performed in August 2022 demonstrated widespread multisystem involvement, including extensive cutaneous thickening with intense FDG uptake involving the scalp, external auditory canals, face, axillae, perineum, and inguinal regions (SUV max 10.4), diffuse periodontal/gingival uptake around the maxilla and mandible extending to the inferior walls of both maxillary sinuses (SUV max 6.1), focal skeletal lesions involving the left petrous temporal bone, left scapula (with lytic destruction), and the right acetabulum (SUV max 4.8), diffuse thyroid uptake (SUV max 13.4), focal hypermetabolism in the descending/sigmoid colon (SUV max 4.8 on delayed imaging), cervical/supraclavicular lymphadenopathy (SUV max 11.2), representative pulmonary cystic lesions with mild uptake (SUV max 1.6) with a concurrent non–FDG-avid solid nodule, and a hypermetabolic nodule in the right obturator internus muscle (SUV max 2.9) (**Figures 3A–H**).

After six cycles of cytarabine-based induction chemotherapy (4), follow-up ^18^F-FDG PET/CT in April 2023 demonstrated near-complete metabolic remission, with previously FDG-avid cutaneous lesions and imaging-suggestive multifocal osseous and extracutaneous involvement (thyroid, colon, and lymph nodes) no longer showing abnormal FDG uptake; only residual low-grade uptake persisted in the perineal skin and maxillomandibular/gingival region (e.g., gingival uptake SUV max 6.1→5.3; perineal uptake SUV max 10.4→3.7), along with mild activity in the mastoid air cells and right obturator internus. Newly noted nasopharyngeal uptake with mildly avid cervical nodes was favored to represent inflammation (**Figures 3I–L**).

**Figure 4:**
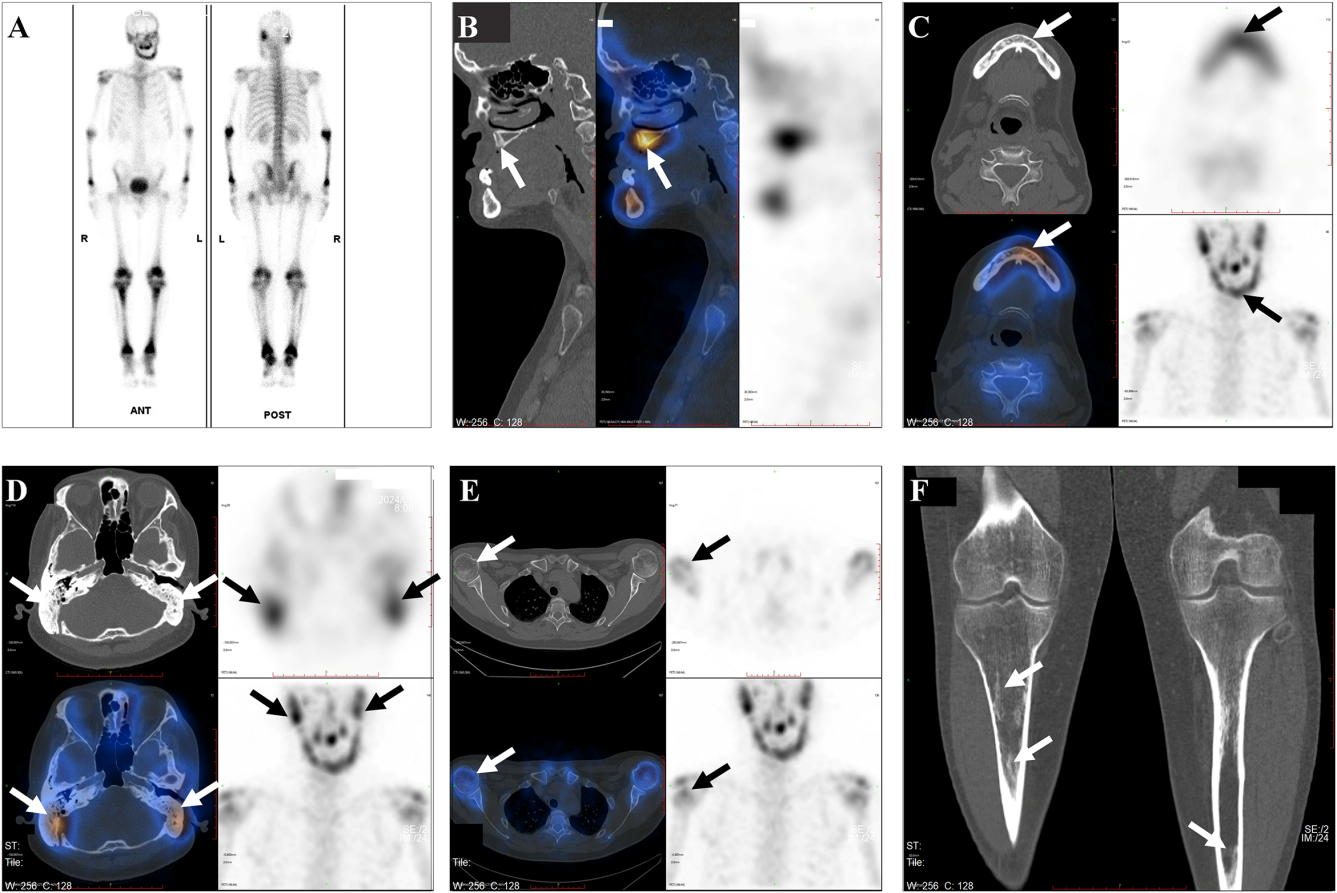
Whole-body ^99m^Tc-MDP bone scintigraphy with SPECT/CT (July 2024) showing multifocal skeletal tracer uptake in adult multisystem Langerhans cell histiocytosis. **(A)** Planar whole-body scintigraphy (anterior/posterior) demonstrates multifocal increased MDP uptake, prominent in the craniofacial region and appendicular skeleton. **(B)** SPECT/CT of the maxilla shows focal cortical thinning/resorption with corresponding MDP uptake. **(C)** SPECT/CT of the mandible demonstrates heterogeneous osseous density/sclerosis with increased uptake. **(D)** SPECT/CT of the temporal/mastoid regions shows uptake with sclerotic change. **(E)** SPECT/CT of the proximal humeri shows multifocal uptake with subtle sclerotic foci. **(F)** Representative extremity CT shows patchy medullary hyperattenuation in the proximal tibia; planar scintigraphy demonstrates increased uptake in the lower legs. Arrows indicate representative abnormalities.

In the months preceding the July 2024 evaluation, the patient developed progressive maxillary/alveolar bone destruction with loss/extraction of multiple upper teeth, prompting a systematic skeletal re-assessment in the context of his prior disseminated disease. In July 2024, whole-body bone scintigraphy with SPECT/CT showed multifocal increased ^99m^Tc-MDP uptake involving the maxillomandibular bones, bilateral temporal bones, elbows/forearms, knees, and lower legs (tibiae and fibulae). SPECT/CT further revealed focal maxillary bone resorption, heterogeneous mandibular sclerosis with tracer accumulation, and increased medullary attenuation in the distal femora and tibiae/fibulae—suggesting ongoing osseous involvement and remodeling (**Figure 4**).

Following multidisciplinary discussion, antiresorptive therapy (zoledronic acid) was initiated for skeletal protection in the setting of multifocal osseous abnormalities, and cytarabine maintenance was continued/extended given the favorable prior response to cytarabine-based induction and institutional practice. Therefore, the patient continued on maintenance therapy with subcutaneous cytarabine administered every 35 days for four additional cycles, along with supportive care. Zoledronic acid was introduced in late 2024 for skeletal protection. In January 2025, treatment was further adjusted at an outside institution to a thalidomide-based combination regimen with corticosteroids and cyclophosphamide. At last contact in May 2025, repeat imaging was recommended; however, the patient did not return for follow-up imaging and was lost to follow-up.

The prolonged diagnostic course was associated with significant anxiety and psychosocial distress; however, the patient reported partial psychological relief after the establishment of a definitive diagnosis and initiation of targeted therapy.

## 3 Discussion

Overall, this case showed pathologically confirmed cutaneous LCH with imaging-suggestive multifocal osseous involvement on ^18^F-FDG PET/CT and whole-body bone scintigraphy with SPECT/CT, together with imaging-suggestive extracutaneous uptake of the thyroid, gastrointestinal tract, lymph nodes, lungs (cystic lesions), and an intramuscular nodule. The constellation of diffuse thyroid hypermetabolism, prominent jaw-periodontal involvement, pulmonary cystic lesions, gastrointestinal hypermetabolism, and a hypermetabolic intramuscular nodule in a single adult patient illustrates a highly disseminated phenotype and highlights the breadth of extracutaneous disease that may be uncovered by whole-body staging. Taken together, this constellation represents an uncommon pattern of multisystem involvement in adult LCH, with several extracutaneous sites occurring concurrently in a single patient. Long-bone disease is within the described skeletal spectrum of LCH; in an adult cohort of biopsy-proven osseous LCH, lower-extremity (“legs”) lesions were reported in ~18% of patients (8), supporting that tibial involvement is compatible with adult disease patterns. In LCH overall (mixed-age cohorts), oral involvement has been reported in ~10% of LCH cases in a large review (9); among skeletal lesions, mandibular involvement accounts for ~7–9% and maxillary involvement for ~1% (10).

Adult-onset multisystem LCH frequently eludes prompt diagnosis due to its clinical heterogeneity. Our patient exemplifies a diagnostically challenging course dominated by two years of recurrent ulcerative intertriginous skin lesions with poor response to empirical therapies, ultimately clarified only after biopsy from the ulcer edge. In adults with LCH, isolated cutaneous disease is uncommon (~5–10%), whereas cutaneous involvement more often occurs as part of multisystem disease (~20–50%) (4). These observations support a practical message that the skin can serve as an accessible “window” to systemic LCH: persistent unexplained ulcerative/exudative lesions—particularly in intertriginous areas—should trigger early biopsy with immunohistochemistry. Importantly, once cutaneous LCH is confirmed in an adult, a systematic staging evaluation (e.g., whole-body ^18^F-FDG PET/CT with organ-directed tests as indicated) is warranted, given the frequent systemic association.

A further instructive aspect of this case is the complementary—and potentially discordant—information provided by serial multimodality imaging (11). While follow-up ^18^F-FDG PET/CT after induction chemotherapy suggested near-complete metabolic remission, ^99m^Tc-MDP whole-body bone scintigraphy with SPECT/CT approximately one year later demonstrated persistent multifocal tracer uptake, most prominent in the craniofacial skeleton and long bones, with corresponding CT changes suggestive of ongoing remodeling (**Figure 4**). This divergence is biologically plausible because FDG uptake primarily reflects metabolically active inflammatory/histiocytic infiltration, whereas MDP uptake reflects osteoblastic activity and bone remodeling and is not disease-specific (12), which may persist after metabolic response or may indicate ongoing low-grade osseous disease not captured by FDG-PET at a given time point. Accordingly, osseous assessment in adult LCH should not rely on FDG-PET alone (13); when symptoms persist, craniofacial disease is prominent, or relapse is suspected, whole-body bone scintigraphy with SPECT/CT provides PET/CT-complementary information on osseous remodeling, and suspected active lesions should be confirmed by MRI and/or histology when feasible.

Limitations of this report include its single-case nature and the predominantly qualitative imaging assessment. Molecular profiling was limited (BRAF p.V600E was negative, and broader MAPK-pathway testing was not available). In addition, extracutaneous FDG uptake was not confirmed by organ-directed imaging or histology and should be interpreted as imaging-suggestive only. Persistent skeletal tracer uptake on bone scintigraphy with SPECT/CT lacked histologic or MRI confirmation; therefore, these findings should be interpreted cautiously as reflecting bone remodeling and/or residual osseous involvement rather than definitive viable disease.

## 4 Conclusion

We report an adult case of multisystem LCH in which ulcerative intertriginous skin lesions served as the initial and most accessible clue, with definitive diagnosis established by biopsy and immunophenotyping. Whole-body ^18^F-FDG PET/CT revealed extensive cutaneous involvement and imaging-suggestive multifocal osseous and extracutaneous uptake. Despite near-complete metabolic remission on post-induction PET/CT, subsequent ^99m^Tc-MDP bone scintigraphy with SPECT/CT demonstrated persistent multifocal osseous activity, highlighting the complementary value of multimodality imaging during longitudinal assessment. Clinically, this case emphasizes the importance of early biopsy for persistent unexplained ulcerative/exudative skin lesions in adults and supports comprehensive staging once cutaneous LCH is confirmed, with organ-directed follow-up imaging tailored to symptoms and suspected skeletal activity.

## Data Availability

The original contributions presented in the study are included in the article/supplementary material. Further inquiries can be directed to the corresponding authors.
